# Scarborough Health Conference

**Published:** 1923-06

**Authors:** 


### SCARBOROUGH HEALTH CONGRESS.

DELEGATES from all over the country, including distinguished visitors from the Continent, attended the Public Health Congress organised by the Royal Institute of Public Health, held at Scarborough from May 16 till May 21. Lord Riddell was president of the conference, and his inaugural address dealt with "The Organisation of Industry." Meeting in five separate sections, members discussed a variety of important subjects closely affecting the well-being of the nation. Housing and overcrowding, the relation of milk supplies to tuberculosis, and the health of the worker as a national asset were among the subjects treated by men and women well qualified to deal with them.

### The Health of the Worker.

Speaking on healthy conditions of work, Sir Thomas Oliver, Professor of Medicine at the Newcastle-on-Tyne College of Medicine, said that the percentage of physical fitness was irregularly distributed throughout the country. For example, he pointed out, the men of Sheffield were much superior to those of Leeds, and the coal miners of the West of Wales were superior to their confrêres in the eastern portion of the Principality. One of the explanations of the superior physique of the Western Welsh coal miner, compared with the native of the Eastern counties, was that the coal miners of the west were the sons of small farmers, that the coal seams had a greater height, and the coal therefore was more easily won. It was the experience of the Ministry of War that the mining class gave the best recruits to the army. In Leeds the large amount of tailoring carried on, often amid unwholesome surroundings, was probably responsible for the quantity of pulmonary disease observed among the workers. A better and more uniform system of recording sickness in our large factories was required.

### Clean Milk.

Considerable time was devoted to questions connected with tuberculosis, which Dr. Jase Walker pointed out was undoubtedly an industrial disease, but for which overcrowding seemed to be less responsible than previously supposed. Seven per cent. of the milk offered for sale in this country is tuberculous, said Mr. Wilfred Buckley, C.B.E., chairman of the National Clean Milk Society, who urged that milk should be standardised—much of that on the market was worthless.

### The Place of the Voluntary Worker.

The place of the voluntary worker in public health was discussed by Mrs. Edwin Gray (York), vice-president of the Yorkshire Federation of Maternity and Child Welfare. She would like to see in all local government divisions some Council of Public Health, which, as occasion required, could bring together for discussion, or to initiate some new beneficent scheme, representatives of all the statutory bodies, and all voluntary associations—doctors, teachers, nurses, health visitors, midwives, and organised bodies of workers such as trade unions and trades and labour councils. Such a council could divide itself into sections for the study of special problems, and be a power to form public opinion.

